# Screen Printed Copper and Tantalum Modified Potassium Sodium Niobate Thick Films on Platinized Alumina Substrates

**DOI:** 10.3390/ma14237137

**Published:** 2021-11-24

**Authors:** Brigita Kmet, Danjela Kuščer, Soma Dutta, Hana Uršič, Aleksander Matavž, Franck Levassort, Vid Bobnar, Barbara Malič, Andreja Benčan

**Affiliations:** 1 Electronic Ceramics Department, Jožef Stefan Institute, 1000 Ljubljana, Slovenia; brigita.kmet@ijs.si (B.K.); danjela.kuscer@ijs.si (D.K.); som@nal.res.in (S.D.); hana.ursic@ijs.si (H.U.); barbara.malic@ijs.si (B.M.); 2 Condensed Matter Physics Department, Jožef Stefan Institute, 1000 Ljubljana, Slovenia; aleksander.matavz@ijs.si (A.M.); vid.bobnar@ijs.si (V.B.); 3 Materials Science Division National Aerospace Laboratories, Bangalore 560017, India; 4 Jožef Stefan International Postgraduate School, 1000 Ljubljana, Slovenia; 5 GREMAN UMR 7347, Université de Tours, CNRS, INSA-CVL, 37200 Tours, France; franck.levassort@univ-tours.fr

**Keywords:** lead-free, KNN, thick film, microstructure, electromechanical properties

## Abstract

We show how sintering in different atmospheres affects the structural, microstructural, and functional properties of ~30 μm thick films of K_0.5_Na_0.5_NbO_3_ (KNN) modified with 0.38 mol% K_5.4_Cu_1.3_Ta_10_O_29_ and 1 mol% CuO. The films were screen printed on platinized alumina substrates and sintered at 1100 °C in oxygen or in air with or without the packing powder (PP). The films have a preferential crystallographic orientation of the monoclinic perovskite phase in the [100] and [−101] directions. Sintering in the presence of PP contributes to obtaining phase-pure films, which is not the case for the films sintered without any PP notwithstanding the sintering atmosphere. The latter group is characterized by a slightly finer grain size, from 0.1 μm to ~2 μm, and lower porosity, ~6% compared with ~13%. Using piezoresponse force microscopy (PFM) and electron backscatter diffraction (EBSD) analysis of oxygen-sintered films, we found that the perovskite grains are composed of multiple domains which are preferentially oriented. Thick films sintered in oxygen exhibit a piezoelectric *d*_33_ coefficient of 64 pm/V and an effective thickness coupling coefficient *k_t_* of 43%, as well as very low mechanical losses of less than 0.5%, making them promising candidates for lead-free piezoelectric energy harvesting applications.

## 1. Introduction

Piezoelectric ceramics are important components of many devices such as sensors, transducers, actuators, and energy harvesters [1]. Environmentally friendly alkali niobate-based materials compete directly with the most commonly used piezoelectric ceramics based on lead zirconate titanate [2,3]. However, processing dense, phase-pure alkali niobate ceramics such as potassium sodium niobate (K_0.5_Na_0.5_NbO_3_, KNN) is challenging due to humidity-sensitive alkali reagents, volatilization of alkalis at high temperatures, and associated formation of unwanted secondary phases, as well as a narrow temperature range during sintering [4]. Due to the miniaturisation requirements of devices, there is a need for lead-free piezo electrics in layered form. However, the fabrication of high-quality thick films is even more demanding, as the possible interaction between the film and the substrate, the higher volatilization of the alkalis due to the higher surface-to-volume ratio in thick film structures, and the clamping effect of the substrate can negatively affect the densification and final electromechanical behaviour of KNN thick films [5]. In contrast to a number of studies on KNN-based bulk ceramics, the effect of processing conditions on the properties of KNN-based thick films prepared by screen printing [6], electrophoretic deposition [7,8,9], tape casting [10,11], hydrothermal methods [12], or pad-printing [13] has been investigated to a much lesser extent. Ways to improve the densification and thus the final functional properties of KNN-based thick films include the selection of the optimal sintering temperature, time and/or atmosphere, chemical modification of the matrix composition, or addition of sintering aids [6,14,15,16,17]. 

In more detail, Pavlič et al. [6] prepared KNN thick films with the addition of potassium sodium germanate (KNN-KNG) sintering aid by screen-printing and varied the local atmosphere during sintering by using a KNN packing powder (PP). The role of PP was to provide the atmosphere enriched with alkali vapors and thus oppose evaporation of alkalis from the film. The PP should contribute to avoiding or at least diminishing the presence of secondary phases, following the established approach in sintering of lead-based ceramics [18]. The films sintered in the presence of PP were phase-pure and exhibited a more uniform microstructure but had lower density compared to the films sintered without any PP. Sintering temperatures of up to 1100 °C were required to obtain films with high relative densities (above 96%). The lower converse piezoelectric *d*_33_ coefficient of the KNN films compared to bulk (82 pm/V versus 138 pm/V at 4 kV/cm) was attributed to the clamping by the substrate, as the latter can elastically constrain the motion of non-180° domain walls under an applied electric field [19].

While it is generally accepted that sintering of polycrystalline oxides in oxygen atmosphere promotes densification [20], the literature on KNN is not consistent. For example, some authors claim that sintering KNN-based ceramics at a low oxygen partial pressure promotes alkali evaporation [21], while others report that the evaporation of alkalis is suppressed [22]. Mercier et al. [14,23] studied the effects of sintering temperature and atmosphere on the properties of Sr-doped KNN thick films prepared by electrophoretic deposition on platinized alumina substrates. As the sintering temperature increased from 1090 to 1110 °C, the relative density increased, which was reflected in an increase of the electromechanical properties. The influence of the sintering atmosphere, i.e., air and oxygen, on the densification and electromechanical properties was not significant, but traces of a polyniobate phase were detected only in oxygen-sintered films. Copper-based additives, including CuO [24] and K_5.4_Cu_1.3_Ta_10_O_29_ (KCT) [25] have been found effective as sintering aids, and they have also contributed to piezoelectric hardening which was attributed to incorporation of copper ions into the B-sites of the perovskite lattice. The mechanical quality factor (*Q_m_*) increased from 90 in KNN to 1300 upon addition of 0.38 mol% KCT [25]. Park et al. [26] reported that the addition of 1 mol% CuO and 0.38 mol% KCT resulted in highly dense ceramic (97% relative density) with very high *Q_m_* of ~3000, *d*_33_ 94 pC/N, k_p_ 0.38, *ε* 285 and *tgδ* 1.8%.

In this study, we investigate thick films of KNN modified with 0.38 mol% KCT and 1 mol% CuO (formulation based on ref. [24], denoted as KNN-KCT-CuO) on Pt/Al_2_O_3_ platinized alumina substrates. Screen printing was selected for thick-film deposition as an established and reliable deposition method that allows us to deposit materials with well-defined dimensions on flat substrates [27]. The KCT and CuO additives were expected to contribute both to densification as well as to piezoelectric hardening of the KNN matrix. With the aim of obtaining dense films with good functional properties, we investigated the influence of the sintering atmosphere, air or oxygen, and the use of PP on the structure and microstructure of KNN-KCT-CuO thick films. We show that 30 μm thick KNN-KCT-CuO films sintered at 1100 °C in oxygen without any PP are dense and exhibit good electromechanical properties, in particular high *Q_m_*, suggesting that they are well suited for energy harvesting applications. 

## 2. Materials and Methods 

By conventional solid-state reaction, the powders of K_0.5_Na_0.5_NbO_3_ (KNN) and K_5.4_Cu_1.3_Ta_10_O_29_ (KCT) were prepared separately from K_2_CO_3_ (99.9%, Chempur, Karlsruhe, Germany), Na_2_CO_3_ (99.9%, Chempur, Karlsruhe, Germany), Nb_2_O_5_ (99.9%, Aldrich, St. Louis, Missouri, MI, USA), Ta_2_O_5_ (99.85%, Alfa Aesar, Karlsruhe, Germany) and CuO (99.7%, Alfa Aesar, Karlsruhe, Germany). The starting carbonate powders were ground and dried at 200 °C for 24 h to remove moisture. All powders were stored in dry atmosphere, weighted in stoichiometric ratio, and milled in acetone for 4 h using a planetary ball mill. After drying, the KNN powder was synthesized by two calcinations at 800 °C and 750 °C for 4 h, and the KCT powder was calcined at 900 °C for 4 h. The mixture of KNN, 0.38 mol% KCT and 1.0 mol% CuO powders was homogenized for 2 h in acetone medium with a planetary mill. The KNN-KCT-CuO paste was prepared from the respective powder mixture by adding an organic vehicle (alpha-terpineol, ≥98%, Merck, Darmstadt, Germany; 2-2-butoxy-ethoxy-ethyl acetate, ≥98%, Merck, Darmstadt, Germany and ethyl cellulose, 48% ethoxyl, Sigma Aldrich, St. Louis, Missouri, MI, USA) in a 60/40 weight ratio on a three-roll mill and screen printed on platinized alumina substrates (platinum paste, E1192, Ferro Corp., Mayfield Heights, Ohio, OH, USA; Al_2_O_3_ substrate, Kyocera A493, Kyoto, Japan). After drying at 150 °C for 15 min the layers were isostatically pressed at 300 MPa and annealed in a tube furnace at 1100 °C for 2 h with an intermediate step at 500 °C for 2 h. The heating rates were 2 K/min to 500 °C and 5 K/min to 1100 °C. The cooling rate was 5 K/min. The samples were sintered in oxygen (marked as KNN-KCT-CuO_O_2_) and in air with and without the packing powder (marked as KNN-KCT-CuO_AIR_PP and KNN-KCT-CuO_AIR, respectively). For the packing powder, 6g of the KNN-KCT-CuO powder was used to sustain the vapour pressure of alkalines in the local atmosphere within the covered crucible with the volume of ~40 mL. 

The KNN-KCT-CuO bulk ceramic was prepared as reference by sintering powder compacts, isostatically pressed with 200 MPa, at 1000 °C for 2h in air with heating and cooling rates of 5 K/min.

The phase composition of the crushed KNN-KCT-CuO ceramic and sintered thick films was investigated by XRD analysis (X’Pert PRO MPD with Cu-K𝛼1 radiation) at room temperature. To obtain a quantitative phase composition, Rietveld refinement was performed using Topas R software (Bruker, AXS, Karlsruhe, Germany). The experimental patterns were fitted against the calculated patterns, based on the monoclinic unit cell with the space group Pm [28]. The scale factor, the unit cell parameters and the crystallite size were refined for each phase. The errors in the quantitative phase analysis did not exceed 5% relative.

Microstructural analysis of the thick-film samples was performed using a field emission scanning electron microscope (JSM 7600F, Jeol, Tokyo, Japan with the resolution of 1.0 nm at 15 kV) equipped with an energy dispersive X-ray spectroscopy system (EDXS, Oxford Instruments, Abingdon, UK) and electron backscatter diffraction (EBSD, Nordlys, Oxford Instruments, Abingdon, UK). Imaging was performed with the secondary (SE) or the backscattered electron detector (BSE) at 15 kV or 20 kV accelerating voltages, at room temperature. Porosity was evaluated by image analysis of cross-sections. Kikuchi diffraction patterns were indexed by KNbO_3_ with orthorhombic symmetry. A medium level of zero solution extrapolation was performed on EBSD orientation map. ImageJ software (National Institute of Mental Health, Bethesda, Maryland, MD, USA) was used to analyze the ratio of individual colours on EBSD images.

Atomic Force Microscope (AFM, Asylum Research Molecular Force Probe 3D, MFP-3D, Santa Barbara, CA, USA) equipped with a piezo-response force mode (PFM) and a Ti/Ir coated silicon tip (Asyelec_01_R2, Oxford Instruments, Wiesbaden, Germany) was used for the morphology analysis of grains and domains at room temperature. The scanning ac electric voltage of 15 V and frequency of 390 kHz was applied during scanning.

Standard metallographic methods (cutting, grounding, polishing) were used to prepare the cross sections of the thick films and surfaces for SEM, AFM/PFM, and EBSD analysis. Prior the SEM analysis, the samples were coated with 3 nm of carbon or chromium using a sputter coater (PECS 682, Gatan, Pleasanton, California, CA, USA) to avoid electron charging.

For electrical measurements, 1.5 mm diameter Cr/Au electrodes were sputtered using a RF -magnetron sputtering system (5Pascal). The capacitance (*C*) and dielectric losses (*tgδ*) of thick films were measured at room temperature using a HP 4284 A Precision LCR Meter in the frequency range from 100 Hz to 1 MHz. The capacitance (*C*) versus temperature curves of the thick films were recorded using an impedance analyzer (HP4192A Precision LCR Meter) (Agilent Technologies Inc., Santa Clara, California, CA, USA) at the frequency of 10 kHz and upon cooling with 2 K/min rate for thick films sintered in oxygen and 1 K/min cooling rate for thick films sintered in air, in the temperature range from 430 °C to 25 °C. The dielectric constant (ε) was calculated from the capacitance data. Ferroelectric hysteresis loops (P-E) were measured using an aixACCT TF Analyser 2000FE system (aixACCT Systems GmbH, Aachen, Germany) equipped with high-voltage amplifier TREK 609E-6 at frequencies of 10 Hz. The samples were poled at 120 °C in silicon oil by applying an external DC electric field of 3 kV/mm for 2 h and then cooled to room temperature using a Keithley 248 high voltage power supply. Electrical measurements were performed 24 h after poling at room temperature. The piezoelectric *d*_33_ coefficient was determined at the frequency of 1 kHz by using a double beam laser interferometer (aixDBLI, aixACCT) (aixACCT Systems GmbH, Aachen, Germany).

The electromechanical parameters of the thick films were deduced from the measurements of the complex electrical impedance as a function of the frequency around the fundamental thickness-mode resonance using HP4395 spectrum analyzer and its impedance test kit (Agilent Technologies Inc., Santa Clara, California, CA, USA). An equivalent electrical circuit model was used to simulate the behavior of the electrical impedance of the samples as a function of frequency for the thickness mode. The model retained is the Krimholtz–Leedom–Matthaei (KLM) scheme [29] where mechanical and dielectric losses were introduced [30]. The structure contains three inert layers (alumina substrate and two electrodes) and one piezoelectric layer (thick film) which were all taken into account for the calculation of this theoretical impedance. Analytical expressions for one piezoelectric layer in free mechanical conditions [31] or on a substrate [32] can be obtained. In our case, with additional layers, numerical calculation was used to calculate theoretical electrical impedance. The dielectric constant at constant strain ($\varepsilon_{33}^{S}$), elastic constant at constant electrical displacement ($c_{33}^{D})$, piezoelectric coefficient (*e*_33_), effective thickness coupling coefficient (*k_t_*) and mechanical losses (*δ_m_*) were deduced with a fitting process for the complex experimental electrical impedance. Additional information about this procedure is given elsewhere [13,33].

## 3. Results and Discussions

**Figure 1:**
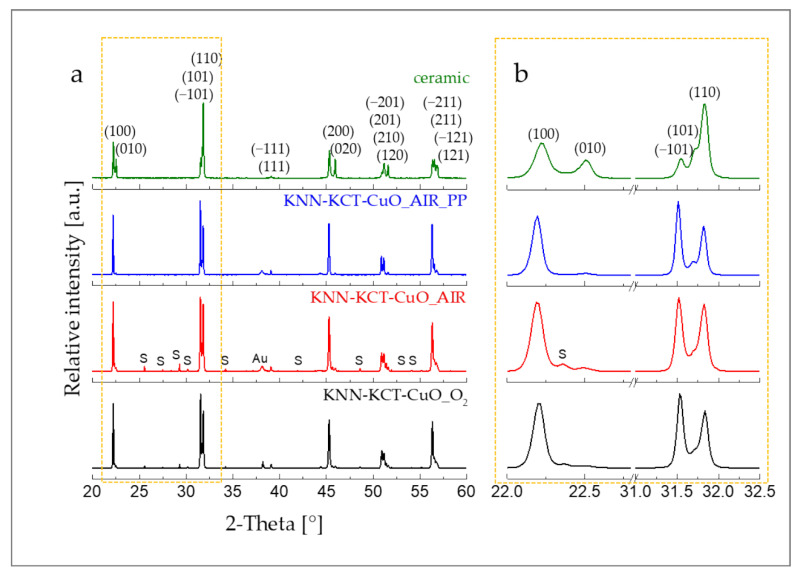
(**a**) XRD patterns of KNN-KCT-CuO thick films sintered at 1100 °C for 2 h in oxygen or in air with and without the packing powder (PP) and KNN-KCT-CuO ceramic sintered at 1000 °C for 2h in air; (**b**) enlarged view of {100} and {110} peaks of the perovskite phase in 2-theta range 22–22.8° and 31–32.5°; (hkl): perovskite phase, S: secondary phase, Au: top gold electrode. Indexing of the perovskite phase is according to the monoclinic unit cell (PDF 61-0319, [28]).

Figure 1a shows the XRD patterns of ~30 μm thick KNN-KCT-CuO films sintered in air with and without PP, and in oxygen without any PP. For comparison the pattern of the ceramic of the same composition, sintered in air, is included. The calculated, observed diffraction patterns, and the differences between the observed and the calculated diffractograms, are shown in Figure S1.

The main phase in all samples is the perovskite phase, which could be indexed with a monoclinic unit cell (PDF 61-0319, [28]). A closer look at the XRD patterns (Figure 1b) reveals that the diffraction peak intensities of the perovskite phase in all three films do not match with those of the ceramic. The intensities of the (100) and (−101) diffraction peaks in the thick films are higher than the respective peaks of the ceramic, suggesting that the films have a preferred crystallographic orientation in the [100] and [−101] directions. The origin of the preferred orientation is related to the domain orientation, as will be further shown by the EBSD analysis. Note that the perovskite diffraction peaks in all thick films are slightly down-shifted compared to the ceramic. The larger perovskite lattice parameters in thick films compared to ceramic could be a consequence of several interdependent factors, such as different incorporation of Ta and Cu into the perovskite due to the different sintering temperature of films and ceramic [34,35], formation of O-vacancies [36] and altered K/Na ratio due to alkali losses [6,37].

Table 1 contains the refined unit cell parameters of the perovskite phase in thick films as determined by the Rietveld refinement method. In all films, the *b* and *β* parameters are similar, with differences being within experimental uncertainty. The *a* and *c* parameters and consequently the unit cell volumes of the films sintered in air and oxygen without the PP are slightly smaller than respective data of the film sintered with the PP. We tentatively relate the unit cell contraction in the films sintered without any PP to the change in the chemical composition of the perovskite phase due to alkali losses. According to Popovič et al. [38], potassium has got a higher vapour pressure over KNN than sodium so it is expected that the Na/K molar ratio in the film increases as a consequence of preferential evaporation of potassium from the film surface upon sintering. It was shown that the unit cell volume decreases with increasing fraction of sodium in K_x_Na_1–x_NbO_3_ solid solutions [28]. 

Low-intensity diffraction peaks of a secondary phase (S) are seen in the thick films sintered in air and O_2_ without any PP (Figure 1a). The secondary phase was indexed with the tetragonal P4/mbm structure, which can correspond to K_5.75_Nb_10.85_O_30_ polyniobate phase (PDF 38-0297) or to Cu_1.3_K_5.4_Ta_10_O_29_ (PDF 43-0334). We note that the fraction of Ta in the KNN-KCT-CuO formulation was below 1%; hence, the polyniobate should be the predominant secondary phase. The amount of the secondary phase in KNN-KCT-CuO_AIR and KNN-KCT-CuO_O_2_ thick films is 9.2 wt% and 7.5 wt%, respectively. The presence of a secondary polyniobate phase, which may form due to segregation of alkali vacancies [37], additionally indicates the deviation from the nominal chemical composition due to increased alkali losses in the thick films sintered without PP.

**Figure 2:**
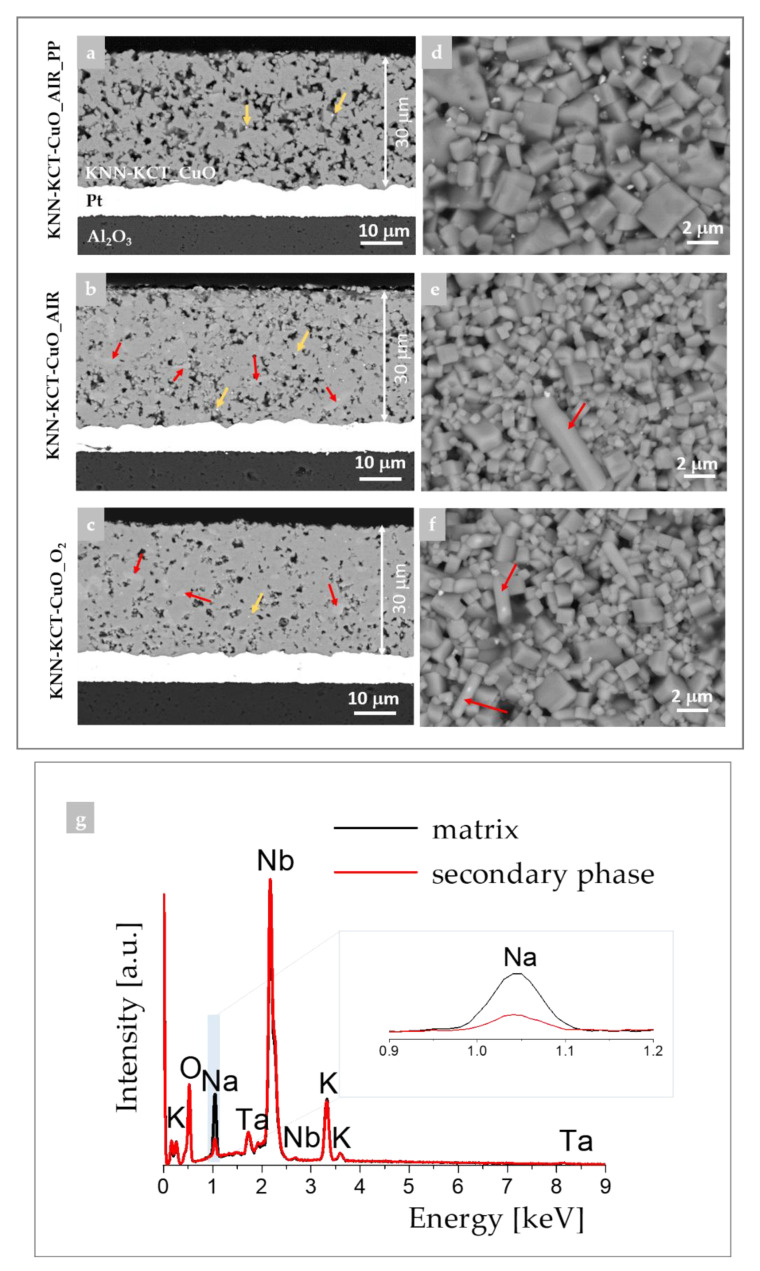
BE-SEM cross-section and surface images of about 30 μm thick KNN-KCT-CuO films sintered (**a**,**d**) in air with PP, (**b**,**e**) in air without PP and (**c**,**f**) in O_2_ without PP. (**g**) EDXS spectra from the matrix (black curve) and from elongated grains of the sodium-depleted secondary phase (red curve) of the KNN-KCT-CuO_O_2_ film. Red arrows indicate elongated grains of the niobium-rich secondary phase, orange arrows indicate the tantalum-rich phase.

Further, we investigated the influence of the sintering atmosphere on the microstructure and phase composition of thick films by SEM. Cross-section and surface micrographs of the thick films are collected in Figure 2 and Figure S2. All films have uniform thicknesses and adhere well to the platinized alumina substrates (Figure 2a–c). The porosity of the thick film sintered in the presence of PP evaluated by image analysis is ~13% (Figure 2a) and is noticeably higher than the porosity of the films sintered without any PP, i.e., ~8% and ~6% in KNN-KCT-CuO_AIR and KNN-KCT-CuO_O_2_, respectively (Figure 2b,c). Thus, a lower fraction of pores in the films sintered without any PP is in agreement with the observation of Pavlič et al. [6] for KNN-KNG films.

Trace amounts of Ta-rich inclusions were found in all thick films (Figure 2a–c—indicated by orange arrows). Elongated grains of the Nb-rich secondary phase were detected only in the films sintered without any PP (Figure 2b,c, indicated by red arrows). According to the EDXS analysis, this Nb-rich phase is sodium-deficient compared to the matrix phase (Figure 2g). The presence of Ta in the matrix phase in the amount of less than 1 at% was confirmed, while the amount of Cu was below the detection limit of the EDXS. The fraction of secondary phases was higher in thick films sintered without PP, consistent with XRD analysis (see Figure 1a).

The images of the surface microstructure reveal cuboidal grains characteristic of KNN [37,39]. The grain size is in the range from a few 0.1 μm to ~3 μm in the film sintered with the PP (Figure 2d) or to ~2 μm in the films sintered without any PP notwithstanding the type of the atmosphere (Figure 2e,f). Elongated grains of Nb-rich inclusions are discerned in these latter films.

**Figure 3:**
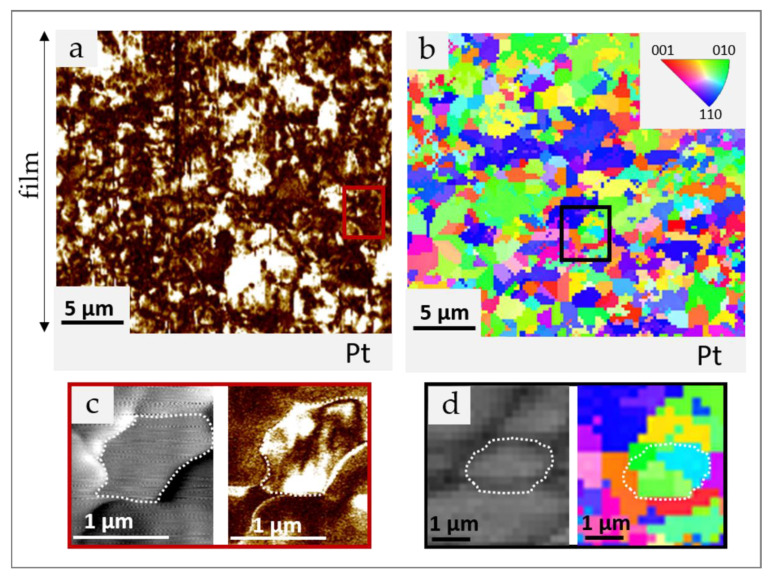
(**a**) Out of plane amplitude PFM image and (**b**) EBSD orientation map with the corresponding colour-key inverse pole figure legend of thick film sintered in O_2_ without any PP in cross sectional view, (**c**) topography and out-of-plane PFM amplitude image, and (**d**) band contrast image and EBSD map showing multidomain grains. Grain boundaries are marked by the white dashed line.

To obtain a deeper insight into the cross-section microstructure, i.e., grain and domain morphology and orientation, we performed the AFM/PFM and EBSD analyses. We report the results for the thick film sintered in oxygen without any PP. The film sintered in air in the presence of the PP contained too much porosity for a reliable analysis. According to the AFM/PFM analysis, no obvious gradient in the grain size and shape is observed through the film thickness (Figure 3a). The grains consist of multiple domains with irregular shapes (Figure 3c). In the EBSD map, indexed with the orthorhombic symmetry (Cm2m symmetry) (Figure 3b), the domains are coloured according to their orientation. The colour occupancy estimation shows that ~26% of the domains are green or blue, only ~ 5% are red, other individual colours are less than 5%, indicating preferential orientation in [010]_ortho_ and [110]_ortho_ directions in the cross-sectional view. The directions [110]_ortho_ and [010]_ortho_ are equivalent to [100]_mono_ and [−101]_mono_ directions, thus EBSD results confirm those from XRD analysis. As shown in Figure 3d, the grains are composed of domains with different orientations. The results reveal that the overall preferred crystallographic orientation of the perovskite phase in thick films detected by XRD (see Figure 1) can be explained by a specific domain configuration and is not due to the orientation at the level of individual grains, i.e., microstructural texturing, as also suggested by Pavlič et al. [6]. Preferential orientation has been attributed to compressive stresses that develop in the film upon cooling due to the thermal expansion mismatch between the KNN film and the alumina substrate and due to stress associated with KNN phase transitions [6].

**Figure 4:**
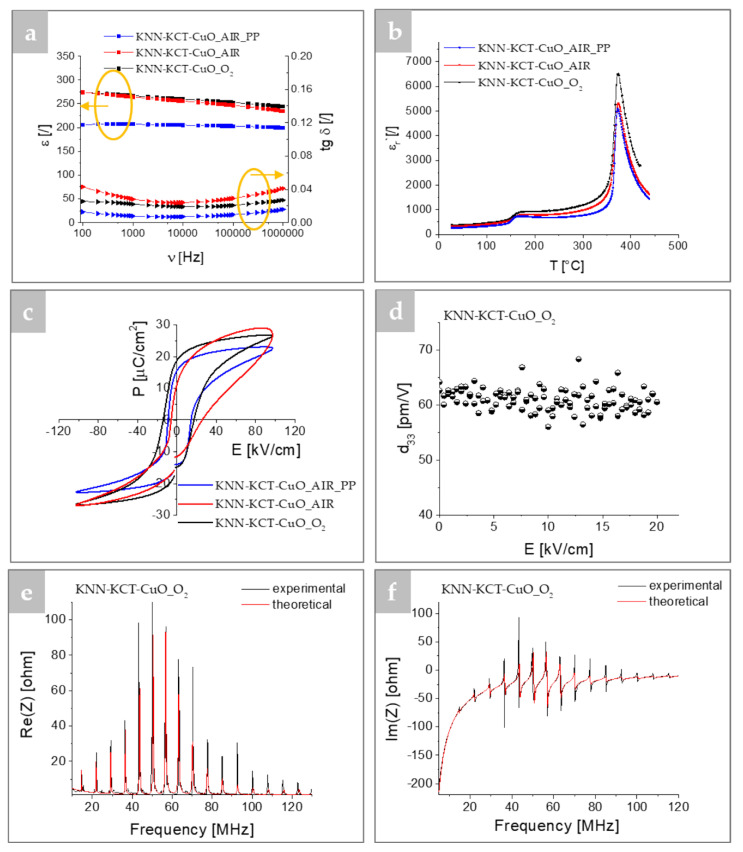
(**a**) Dielectric constant and dielectric losses as a function of frequency at room temperature, (**b**) ε_r_ as a function of temperature measured at 10 kHz and (**c**) polarization hysteresis loops (P-E) measured at room temperature at 10 Hz for thick films sintered in oxygen or in air with and without packing powder. (**d**) *d*_33_ values as a function of dc electric field, (**e**) real and (**f**) imaginary parts of the electrical input impedance as a function of frequency around the fundamental resonance (black line—experimental, red line—theoretical) for the thick film sintered in oxygen, measured at room temperature.

Furthermore, we investigated the influence of the sintering atmosphere on the dielectric, ferroelectric, and electromechanical properties of the ~30 μm thick films (Figure 4 and Figure S3 and Table S2). Figure 4a shows the dielectric constants and losses of KNN-KCT-CuO thick films measured in air from 100 Hz to 1 MHz. At 10 kHz, the dielectric constant of the thick film sintered in air with PP is ~205, while the values of the films sintered in oxygen and air without any PP are higher, i.e., 260 and 256, respectively. The lower dielectric constant of the film sintered with PP may be due to the higher porosity of the film compared to the films sintered without any PP. For all three films, the dielectric constant decreases with increasing frequency, although the decrease is only slight for the film sintered in air with PP and more pronounced for the films sintered without any PP. The dielectric losses at 10 kHz are the lowest for the thick film sintered in air in the presence of the PP (0.01), compared to the thick films sintered in air or in oxygen without any PP, i.e., 0.03 and 0.02, respectively. We relate the lower loss to lower conductivity of the KNN-KCT-CuO_AIR_PP film. The films sintered in the presence of the PP have only traces of Ta-rich inclusions and we assume that in comparison to the films sintered without any PP they contain less crystalline defects such as A-site vacancies. Namely, it is known from the literature that increasing the concentration of A-site vacancies in the KNN-lattice promotes the formation of secondary niobium-rich phase [37], which has been observed in films sintered without PP.

The temperature dependence of the dielectric constant for all three thick films at 10 kHz reveals two peaks at ~374 °C and ~172 °C, marking the phase transitions from cubic to tetragonal (T_c_) and from tetragonal to monoclinic (T_T–M_), respectively, regardless of the use of PP or the sintering atmosphere (Figure 4b). A slight decrease in both transition temperatures of the films is observed compared to KNN-KCT-CuO bulk ceramics (T_C_ ~ 406 °C and T_T–M_ ~194 °C) [36]. This decrease could be related to many parameters, such as substrate confinement, grain size, residual stresses, and defect chemistry [16,40]. 

The polarization hysteresis (P-E) loops of the thick films sintered in air with PP and in O_2_ are well-saturated while the film sintered in air without any PP has an asymmetric loop with rounded edges, indicating a contribution of the leakage current (Figure 4c). The oxygen-sintered film exhibits the highest values of remanent polarization (*P_r_*) and coercive field (*E_c_*), 18.5 μC/cm^2^ and 17.5 kV/cm, respectively. The *E_c_* value is almost two times higher than the value reported for KNN-KCT ceramic (9.8 kV/cm) [25] suggesting an enhanced ferroelectric hardness of the film.

The oxygen-sintered film was selected for further electromechanical characterization. As shown in Figure 3d, the converse piezoelectric *d*_33_ coefficient is about 64 pm/V and is relatively independent of the dc electric field. The obtained *d*_33_ value is in the range of reported values for KNN-based thick films modified with Li, Mn, Sr, i.e., 60–150 pC/N. However, a direct comparison is difficult since the obtained values depend on the measurement methods, processing, sintering conditions as well as the film thickness [10,11,14,41].

Experimental and theoretical complex electrical impedances of the KNN-KCT-CuO_O_2_ thick films as function of frequency around the fundamental resonance are collected in Figure 4e,f. The theoretical electrical impedance was derived using the KLM model, which takes into account the acoustic properties of platinum and alumina [14]. The peaks in the real and imaginary parts of the impedance around the fundamental resonance are due to the coupling of the resonance of the thick film with the platinum electrode and the alumina substrate. The theoretical and experimental values of the electrical impedance agree well. The derived electromechanical quantities are included in Table 2. Of note is the effective thickness coupling coefficient *k_t_* of 43% and mechanical losses *δ_m_* which are below 0.5%. The low *δ_m_* (or high *Q_m_*) is related to the hardening effect of the CuO and KCT sintering additives [25]. 

The comparison of electromechanical quantities of KNN-KCT-CuO_O_2_ thick film with donor (Sr) doped KNN film, both sintered at 1100 °C [14] reveals that the dielectric constant at constant strain $\varepsilon_{33}^{S}$, the piezoelectric coefficient *e*_33_, elastic constant at constant displacement $c_{33}^{D}$, and *k_t_* of KNN-KCT-CuO_O_2_ film are higher, while the *δ_m_* is significantly lower compared to the values reported for Sr-doped KNN thick film, see Table 2. Clearly the donor doping differently effects the electromechanical properties of KNN films compared to KCT-CuO, which is reflected in their properties. But an additional role of CuO and KCT as sintering additives to KNN is in enhancing the densification of the thick film. The porosity of the Sr-doped KNN film which was processed without any sintering aids is about 20%, while the porosity of KNN-KCT-CuO is about 6%. It is notable that a high *Q_m_* and low porosity of KCT-CuO modified KNN thick films are advantageous for vibrational energy harvesting applications [42].

## 4. Conclusions

Thick films of KNN with K_5.4_Cu_1.3_Ta_10_O_29_ (KCT) and CuO additives were prepared on platinized alumina substrates by screen-printing and sintering. The atmosphere upon sintering was found to have an important effect on the phase composition and microstructure of the ~30 μm thick KNN-KCT-CuO films. While the presence of the inherent packing powder upon sintering in air resulted in phase-pure perovskite films (according to XRD analysis), the fraction of porosity was high, ~13%. Sintering without any packing powder in air or in oxygen yielded films which contained some amounts of secondary phases. The latter were alkali-poor in comparison to the matrix phase, indicating evaporation of alkalis from the films. Oxygen-sintering contributed to a slightly lower fraction of porosity, about 6%, than air-sintering. Of particular note are the good electromechanical properties of the KNN-KCT-CuO thick films sintered in oxygen. The low mechanical losses of less than 0.5% are a consequence of the hardening effect of the KCT-CuO additives. The present work highlights the importance of controlling the sintering conditions in thick-films processing and suggests further work on the topic of lead-free piezoelectric thick films for energy harvesting applications.

## Figures and Tables

**Table 1:** Unit cell parameters of KNN-KCT-CuO thick films.

Sample	*a* (Å)	*b* (Å)	*c* (Å)	*β* (°)	*V* (Å^3^)
KNN-KCT_CuO_AIR_PP	4.0044(4)	3.9482(4)	3.9986(6)	90.302(9)	63.21(1)
KNN-KCT_CuO_AIR	4.0040(4)	3.9482(4)	3.9967(6)	90.300(9)	63.18(1)
KNN-KCT_CuO_O_2_	4.0034(3)	3.9485(5)	3.9967(6)	90.290(9)	63.17(1)

**Table 2:** Electromechanical properties of KNN-KCT-Cu_O_2_ thick films compared to literature data on KNN-based thick films.

Sample	*ρ* (%)	$\varepsilon_{33}^{S}$	$c_{33}^{D}$ **(GPa)**	*e*_33_ (C/m^2^)	*k_t_* (%)	*δ_m_* (%)
KNN-KCT_CuO_O_2_ KNN doped with 0.5 mol% Sr [14]	~94 * 81 ± 3	240 120	140 75	7.4 3.4	43 40	<0.5 16

*ρ*: relative density (*: value obtained from the image analysis of porosity), $\varepsilon_{33}^{S}$: dielectric constant at constant strain, $c_{33}^{D}$*:* elastic constant at constant electrical displacement, *e*_33_ piezoelectric coefficient, *k_t_*: effective thickness coupling coefficient, *δ_m_*: mechanical losses. All measurements were performed at room temperature.

## Data Availability

Not applicable.

## Supplementary Materials

The following are available online at https://www.mdpi.com/article/10.3390/ma14237137/s1, Figure S1: Observed, calculated, and difference profiles for the Rietveld refinement of (a) ceramic, (b) thick film sintered in air with packing powder, (c) thick film sintered in air without packing powder, and (d) thick film sintered in oxygen without packing powder. Vertical bars denote reflection positions. P: perovskite phase, S: secondary phase, Au: top gold electrode, Figure S2: BE-SEM cross-section and surface images of thick KNN-KCT-CuO films sintered (a,d) in air with PP, (b,e) in air without PP and (c,f) in oxygen without PP showing rounded inclusions of tantalum-rich secondary phase (marked by orange arrows) and elongated grains of niobium-rich secondary phase (marked by red arrows), Figure S3: Temperature dependence of the dielectric constant ε_r_ and Curie-Weiss fits of the 10 kHz experimental data above and below cubic-tetragonal phase transition of the thick film sintered (a,b) in air with PP, (c,d) in air without PP and (e,f) in oxygen without PP. The solid lines in (b,d,f) represent fits to the inverse Curie–Weiss law $1/\varepsilon `=\frac{T-\mathrm{To}}{C}$ above and below phase transition temperature, Table S1: Refined structural parameters for ceramic and thick films with (hkl) Miller indices, distances between successive layers of atoms and 2-theta range, Table S2: Phase transition temperatures and Curie–Weiss constants for KNN-KCT-CuO thick films sintered in air with or without PP and in oxygen without PP.
