# Supplementary material for: Screen Printed Copper and Tantalum Modified Potassium Sodium Niobate Thick Films on Platinized Alumina Substrates
Source: Materials (Basel). 2021 Nov 24;14(23):7137. doi: 10.3390/ma14237137 (PMC8658548; doi:10.3390/ma14237137)
Supplement: Supplementary file 1 [file materials-14-07137-s001.zip › materials-1457835-The newest version of supplementary material.pdf]

materials

MDPI

Supplementary Materials

Screen Printed Copper and Tantalum Modified Potassium Sodium Niobate Thick Films on Platinized Alumina Substrates

Brigita Kmet [1,4], Danjela Kuščer [1,4], Soma Dutta [1,3], Hana Uršič [1,4], Aleksander Matavž [2,†], Franck Levassort [5], Vid Bobnar [2,4], Barbara Malič [1,4] and Andreja Benčan [1,4,*]

[1] Electronic Ceramics Department, Jožef Stefan Institute, 1000 Ljubljana, Slovenia; brigita.kmet@ijs.si (B.K.); danjela.kuscer@ijs.si (D.K.); som@nal.res.in (S.D.); hana.ursic@ijs.si (H.U.); barbara.malic@ijs.si (B.M.)
[2] Condensed Matter Physics Department, Jožef Stefan Institute, 1000 Ljubljana, Slovenia; aleksander.matavz@ijs.si (A.M.); vid.bobnar@ijs.si (V.B.)
[3] Materials Science Division National Aerospace Laboratories, Bangalore 560017, India
[4] Jožef Stefan International Postgraduate School, 1000 Ljubljana, Slovenia
[5] GREMAN UMR 7347, Université de Tours, CNRS, INSA-CVL, 37200 Tours, France; franck.levassort@univ-tours.fr
* Correspondence: andreja.bencan@ijs.si; Tel.: +386-1-477-3256
† Currently at cMACS, KU Leuven, Belgium.

Citation: Kmet, B.; Kuščer, D.; Dutta, S.; Uršič, H.; Matavž, A.; Levassort, F.; Bobnar, V.; Malič, B.; Benčan, A. Screen Printed Copper and Tantalum Modified Potassium Sodium Niobate Thick Films on Platinized Alumina Substrates. *Materials* **2021**, *14*, 7137. https://doi.org/10.3390/ma14237137

Academic Editor(s): Radu Robert Piticescu

Received: 26 October 2021
Accepted: 19 November 2021
Published: 24 November 2021

Publisher's Note: MDPI stays neutral with regard to jurisdictional claims in published maps and institutional affiliations.

Copyright: © 2021 by the authors. Licensee MDPI, Basel, Switzerland. This article is an open access article distributed under the terms and conditions of the Creative Commons Attribution (CC BY) license (http://creativecommons.org/licenses/by/4.0/).

Supplement S1. Rietveld refinement for KNN-KCT-CuO_AIR_PP, KNN_KCT-CuO_AIR, KNN-KCT-CuO_O2 thick films and KNN-KCT-CuO ceramic.

Rietveld refinement was performed using Topas R software (Bruker, AXS, Karlsruhe, Germany). The experimental patterns (blue) were fitted against the calculated patterns (red), based on the monoclinic unit cell with the space group Pm.

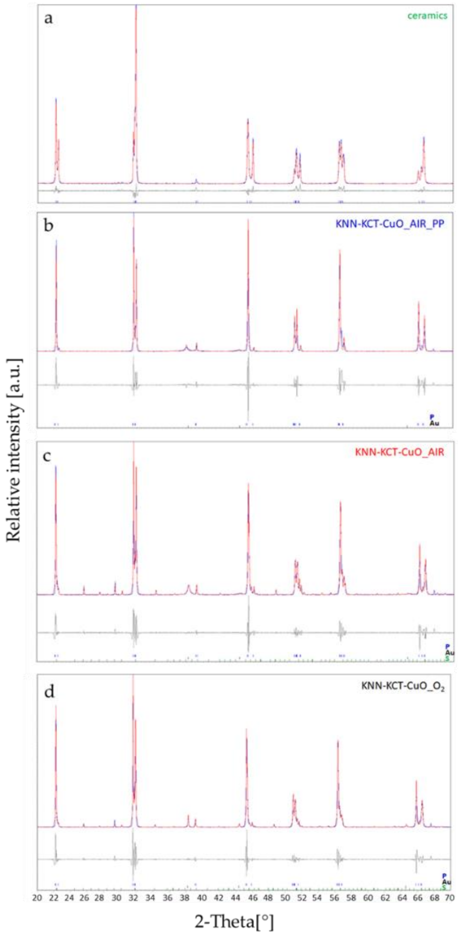

Figure S1. Observed, calculated and difference profiles for the Rietveld refinement of (**a**) ceramic, (**b**) thick film sintered in air with packing powder, (**c**) thick film sintered in air without packing powder and (**d**) thick film sintered in oxygen without packing powder. Vertical bars denote reflection positions. P: perovskite phase, S: secondary phase, Au: top gold electrode.

Table S1. Refined structural parameters for ceramic and thick films with (hkl) Miller indices, distances between successive layers of atoms and 2-theta range.

Ceramic					KNN-KCT-CuO_AIR_PP				
**H**	**K**	**L**	**d**	**2Theta**	**H**	**K**	**L**	**d**	**2Theta**
-1	0	0	4.00235	22.193	-1	0	0	4.00434	22.182
1	0	0	4.00235	22.193	1	0	0	4.00434	22.182
0	0	1	3.99535	22.232	0	0	1	3.99854	22.214
0	1	0	3.9494	22.494	0	1	0	3.9482	22.501
-1	0	1	2.8348	31.534	-1	0	1	2.83693	31.51
1	0	1	2.82047	31.699	1	0	1	2.82202	31.681
-1	1	0	2.81118	31.806	-1	1	0	2.81144	31.803
1	1	0	2.81118	31.806	1	1	0	2.81144	31.803
0	1	1	2.80875	31.835	0	1	1	2.80943	31.827
-1	1	1	2.30296	39.082	-1	1	1	2.30386	39.066
1	1	1	2.29526	39.219	1	1	1	2.29585	39.208
-2	0	0	2.00117	45.278	-2	0	0	2.00217	45.254
2	0	0	2.00117	45.278	2	0	0	2.00217	45.254
0	0	2	1.99767	45.362	0	0	2	1.99927	45.323
0	2	0	1.9747	45.919	0	2	0	1.9741	45.934
-2	0	1	1.79292	50.889	-2	0	1	1.79407	50.854
-1	0	2	1.79103	50.946	-1	0	2	1.7925	50.901
2	0	1	1.78566	51.11	2	0	1	1.78651	51.084
-2	1	0	1.78509	51.128	-2	1	0	1.78569	51.11
2	1	0	1.78509	51.128	2	1	0	1.78569	51.11
1	0	2	1.78379	51.168	1	0	2	1.78497	51.132
0	1	2	1.78261	51.204	0	1	2	1.78363	51.173
1	2	0	1.77089	51.568	1	2	0	1.77063	51.576
-1	2	0	1.77089	51.568	-1	2	0	1.77063	51.576
0	2	1	1.77028	51.587	0	2	1	1.77012	51.592
-2	1	1	1.63257	56.307	-2	1	1	1.63335	56.277
-1	1	2	1.63114	56.36	-1	1	2	1.63217	56.322
2	1	1	1.62708	56.514	2	1	1	1.62764	56.492
1	1	2	1.62567	56.567	1	1	2	1.62647	56.537
-1	2	1	1.62033	56.77	-1	2	1	1.62039	56.768
1	2	1	1.61764	56.873	1	2	1	1.6176	56.875
-2	0	2	1.4174	65.839	-2	0	2	1.41847	65.783
2	0	2	1.41024	66.216	2	0	2	1.41101	66.175
**KNN-KCT-CuO_AIR**					**KNN-KCT-CuO_O$_2$**				
**H**	**K**	**L**	**d**	**2Theta**	**H**	**K**	**L**	**d**	**2Theta**
-1	0	0	4.00395	22.184	-1	0	0	4.00335	22.187
1	0	0	4.00395	22.184	1	0	0	4.00335	22.187
0	0	1	3.99665	22.225	0	0	1	3.99665	22.225
0	1	0	3.9482	22.501	0	1	0	3.9485	22.5
-1	0	1	2.83607	31.52	-1	0	1	2.83561	31.525
1	0	1	2.82126	31.69	1	0	1	2.82129	31.689
-1	1	0	2.8113	31.805	-1	1	0	2.8112	31.806
1	1	0	2.8113	31.805	1	1	0	2.8112	31.806
0	1	1	2.80877	31.834	0	1	1	2.80888	31.833
-1	1	1	2.3034	39.074	-1	1	1	2.30321	39.078
1	1	1	2.29544	39.215	1	1	1	2.29552	39.214
-2	0	0	2.00197	45.259	-2	0	0	2.00167	45.266

2	0	0	2.00197	45.259	2	0	0	2.00167	45.266
0	0	2	1.99832	45.346	0	0	2	1.99832	45.346
0	2	0	1.9741	45.934	0	2	0	1.97425	45.931
-2	0	1	1.79373	50.864	-2	0	1	1.79339	50.874
-1	0	2	1.79176	50.924	-1	0	2	1.79158	50.929
2	0	1	1.78622	51.093	2	0	1	1.78614	51.096
-2	1	0	1.78555	51.114	-2	1	0	1.78536	51.12
2	1	0	1.78555	51.114	2	1	0	1.78536	51.12
1	0	2	1.78428	51.153	1	0	2	1.78435	51.151
0	1	2	1.78296	51.194	0	1	2	1.78299	51.193
1	2	0	1.77059	51.577	1	2	0	1.77065	51.575
-1	2	0	1.77059	51.577	-1	2	0	1.77065	51.575
0	2	1	1.76996	51.597	0	2	1	1.77007	51.594
-2	1	1	1.63309	56.287	-2	1	1	1.63286	56.296
-1	1	2	1.6316	56.343	-1	1	2	1.63149	56.347
2	1	1	1.62742	56.501	2	1	1	1.62738	56.502
1	1	2	1.62595	56.556	1	1	2	1.62602	56.554
-1	2	1	1.62023	56.774	-1	2	1	1.62023	56.774
1	2	1	1.61746	56.88	1	2	1	1.61754	56.877
-2	0	2	1.41803	65.806	-2	0	2	1.4178	65.818
2	0	2	1.41063	66.195	2	0	2	1.41065	66.194

Supplement S2. SEM images

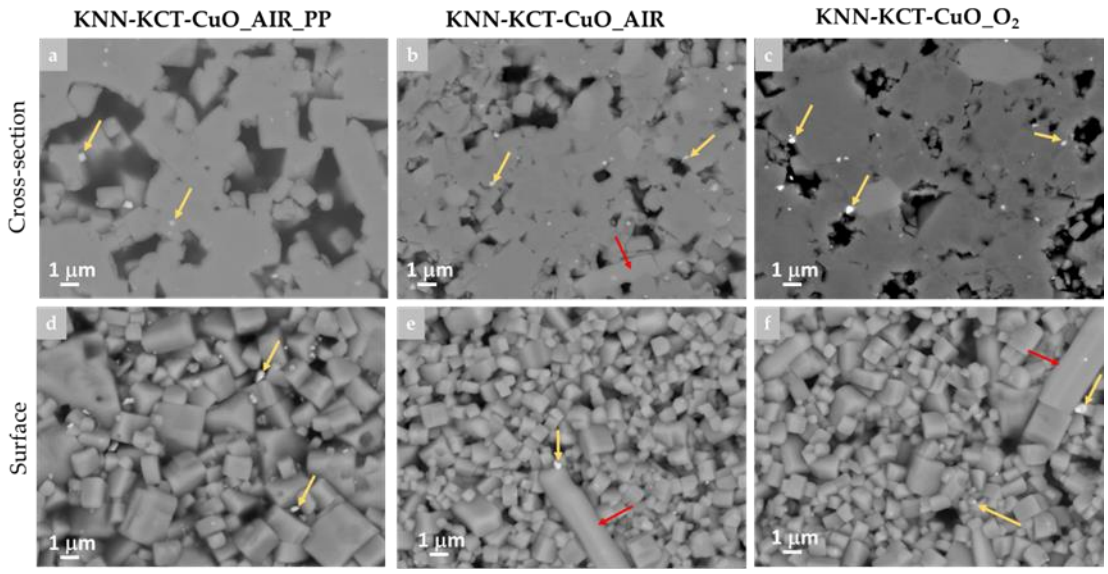

Figure S2. BE-SEM cross-section and surface images of thick KNN-KCT-CuO films sintered (a,d) in air with PP, (b,e) in air without PP and (c,f) in oxygen without PP showing rounded inclusions of tantalum-rich secondary phase (marked by orange arrows) and elongated grains of niobium-rich secondary phase (marked by red arrows).

The porosity was evaluated from the cross-section SEM images, i.e. from the area of ~1500 µm^2 using the programs for image analysis (Corel Photo Paint, Paint Shop Pro and Image Tool). The average porosity with st.dev for KNN-KCT-CuO_AIR_PP, KNN-KCT-CuO_AIR, and KNN-KCT-CuO_O$_2$ are 12. 6 +/- 1.5 %, 8.6 +/ 0.9 %, and 5.8 +/- 1.8 %, respectively.

Supplement S3. Phase transition

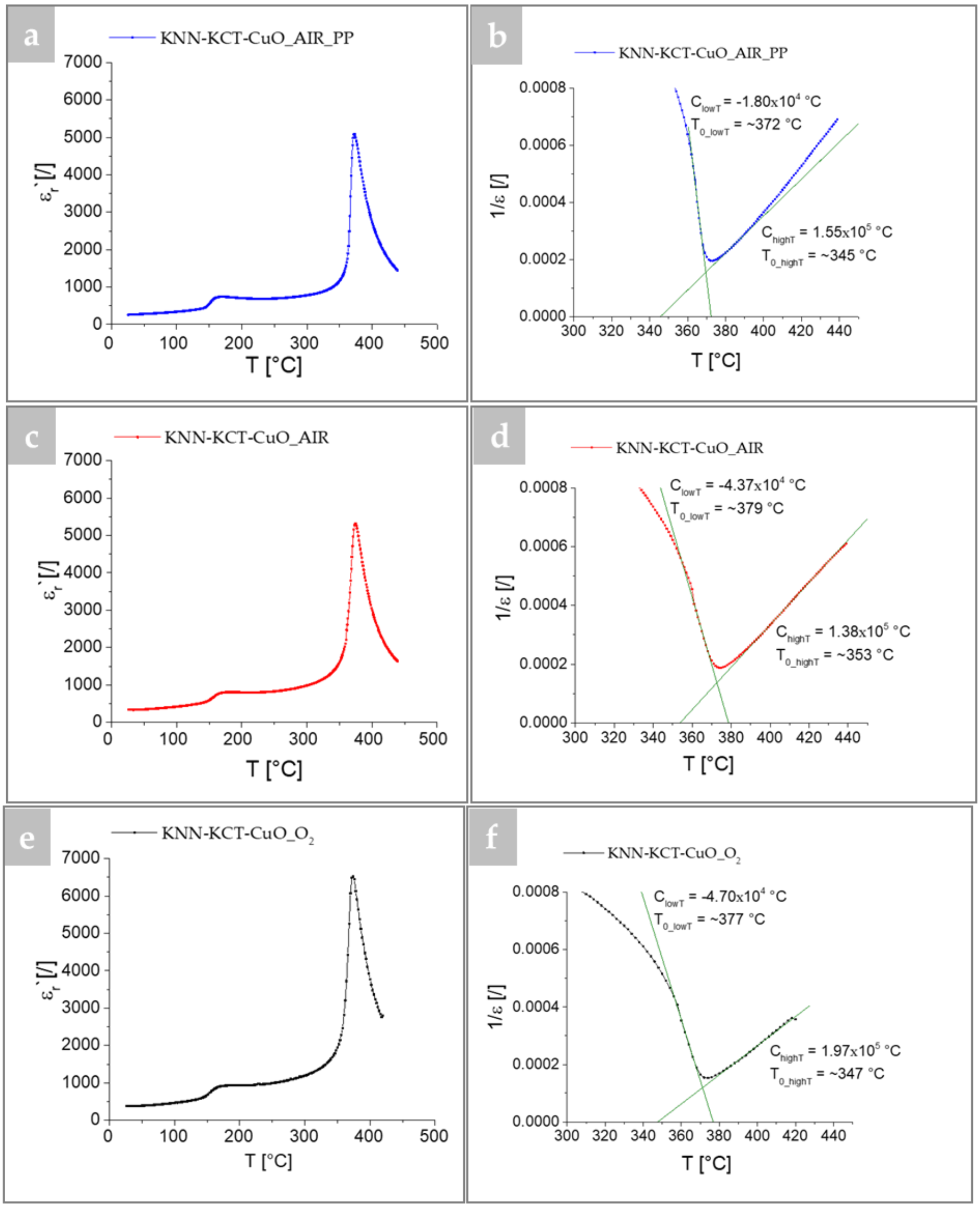

Figure S3. Temperature dependence of the dielectric constant ε_r and Curie-Weiss fits of the 10 kHz experimental data above and below cubic-tetragonal phase transition of the thick film sintered (a,b) in air with PP, (c,d) in air without PP and (e,f) in oxygen without PP. The solid lines in (b,d,f) represent fits to the inverse Curie-Weiss law $1/\varepsilon` = \frac{T-To}{C}$ above and below phase transition temperature.

Table S2. Phase transition temperatures and Curie-Weiss constants for KNN-KCT-CuO thick films sintered in air with or without PP and in oxygen without PP.

**Scheme 105.**	T_c (°C)	$T_{T\text{-}M}$ (°C)	C_{highT} x 10^5 (°C)	T_{0_highT} (°C)	-C_{lowT} x 10^4 (°C)	T_{0_lowT} (°C)
KNN-KCT_CuO_AIR_PP	373	167	1.55	~345	1.80	~372
KNN-KCT_CuO_AIR	374	172	1.38	~353	4.37	~379
KNN-KCT_CuO_O_2	374	172	1.97	~347	4.70	~377

T_c: phase transition temperature from cubic to tetragonal, $T_{T\text{-}M}$: phase transition temperature from tetragonal to monoclinic, T_{o_highT}, C_{highT}, and T_{o_lowT}, C_{lowT}: extrapolated temperature and calculated Curie-Weiss constant using Curie-Weiss law above and below the phase transition temperature, respectively.
